# Application of artificial intelligence large language models in drug target discovery

**DOI:** 10.3389/fphar.2025.1597351

**Published:** 2025-07-08

**Authors:** Xinyu Liu, Jia Zhang, Xiaoran Wang, Maoda Teng, Guoying Wang, Xiaoming Zhou

**Affiliations:** ^1^ Shandong Provincial Hospital Affiliated to Shandong First Medical University, Jinan, China; ^2^ Linyi Central Hospital, Dezhou, China; ^3^ The Second People’s Hospital of Dongying, Dongying, China; ^4^ Department of Research, Shandong Provincial Hospital Affiliated to Shandong First Medical University, Jinan Shandong, China; ^5^ Department of Pharmacy, Dongying People’s Hospital, Dongying, Shandong, China

**Keywords:** large language model, drug target discovery, bioinformatics, multi-omics integration, protein structure prediction

## Abstract

Drug target discovery is a fundamental aspect of contemporary drug research and development. However, the use of conventional biochemical screening, omics analysis, and related approaches is constrained by substantial technical complexity and significant resource requirements. With the advancement of artificial intelligence-based large language models, notable progress has been achieved in drug target identification. During target mining, large language models with natural language comprehension capabilities can efficiently integrate literature data resources and systematically analyze disease-associated biological pathways and potential targets. Notably, models specifically designed for biomolecular “language” have demonstrated advantages across multiple aspects. The genomics-focused large language model has significantly enhanced the accuracy of pathogenic gene variant identification and gene expression prediction. In transcriptomics, large language models enable comprehensive reconstruction of gene regulatory networks. In proteomics, advancements have been made in protein structure analysis, function prediction, and interaction inference. Additionally, the single-cell multi-omics large language model facilitates data integration across different omics technologies. These technological advancements provide multi-dimensional biological evidence supporting drug target discovery and contribute to a more efficient screening process for candidate targets. The development of these models is generally based on deep neural networks of Transformer architecture, and powerful representation capabilities are obtained through large-scale unsupervised pre-training (such as mask language modeling, autoregressive prediction) combined with task-specific supervised fine-tuning. This review systematically examines recent advancements in the application of large language models in drug target discovery, emphasizing existing technical challenges and potential future research directions.

## 1 Introduction

Drug development is characterized by an extended timeline, substantial costs, and considerable risk. From initial research to final product approval, the process typically spans nearly a decade and requires an investment exceeding two billion US dollars (Hinkson et al., 2020). This progression encompasses target identification, candidate compound screening and optimization, preclinical evaluation, clinical trials, and commercial application. Each stage demands extensive resources, exhibits a low success rate, and presents significant industry challenges. As a fundamental stage in research and development, drug target identification plays a decisive role in project success. The primary objective of this process is to identify biological molecules or cellular pathways that serve as key regulators in disease pathogenesis and determine therapeutic intervention points, which include biological macromolecules such as gene loci and membrane receptors. The identification of innovative drug targets forms the basis of the modern drug research and development system, enhancing treatment precision and minimizing adverse effects.

Drug target discovery faces significant challenges due to technical complexities, high resource demands, and intricate disease mechanisms. As of 2022, the number of empirically validated drug targets worldwide remained below 500 (Zhou et al., 2022), highlighting the urgent need to enhance target discovery efficiency, with technological innovation being a critical factor. Currently, mainstream technical strategies include experimental-based approaches, multi-omics integrated analysis, and computer-aided prediction methods (Pun et al., 2023). Experimental-based techniques, such as small molecule affinity probe labeling, play a crucial role in target validation. Multi-omics strategies integrate diverse omics data to identify potential targets; however, these methods require high-quality samples and substantial resources. For example, CRISPR-based functional genome screens can systematically identify essential genes, but their large-scale application is limited by experimental throughput and cost. Computer-aided prediction methods offer potential for identifying novel molecular targets based on the chemical properties of compounds, yet their applicability is restricted by the reliance on three-dimensional protein structural information. Between 2013 and 2022, the median cost and duration of new drug development increased steadily, with median costs reaching about 2.4 billion US dollars—about a 20% rise compared to a decade earlier—and development timelines extending by one to 2 years. Advancing target discovery and achieving technological breakthroughs are essential for improving research and development efficiency.

**FIGURE 1:**
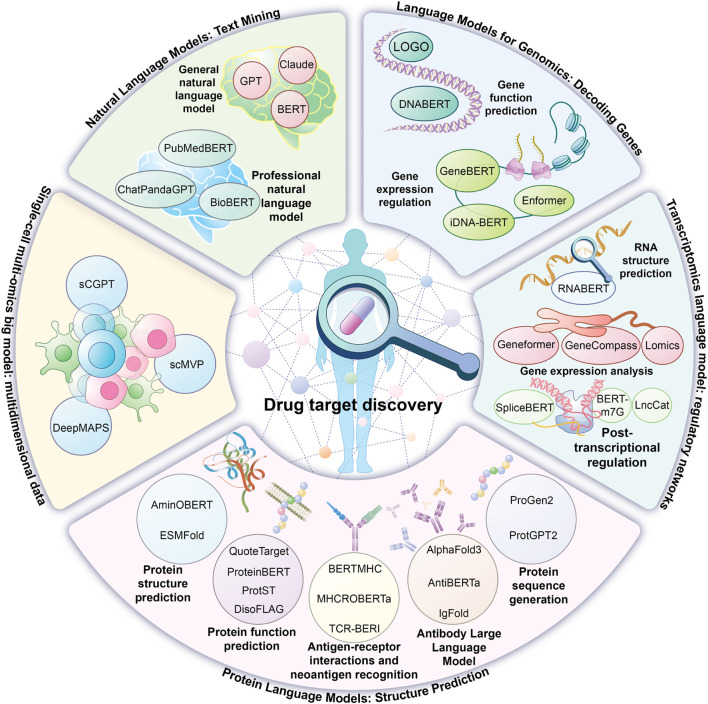
Application of AI big language models in drug target discovery.

Artificial Intelligence (AI), recognized as a transformative technology of the 21st century, has achieved significant advancements in computer vision and natural language processing while also reshaping the entire innovation process in drug research and development (Gangwal et al., 2024). Insilico Medicine, an innovative company leveraging AI to accelerate drug discovery, has developed an “end-to-end” AI platform (PandaOmics + Chemistry42) that has demonstrated high efficiency in drug-target identification and preclinical candidate screening. For instance, in the case of idiopathic pulmonary fibrosis, AI platforms facilitated new target discovery, enabled the launch of the first AI-generated drug, and advanced it to phase II clinical trials within 18 months. Similarly, for HCC treatment, PandaOmics identified CDK20 as a novel target, and in combination with AlphaFold-predicted structures, Chemistry42 generated a novel inhibitor, ISM042-2–048 (IC50 = 33.4 nmol/L), validating the AI platform’s “end-to-end” capabilities (Ren et al., 2023). These practical applications highlight AI’s advantages in enhancing target screening accuracy, reducing drug development timelines, and optimizing research and development efficiency, providing an innovative technical pathway for addressing complex diseases. The emergence of ChatGPT has driven widespread adoption of artificial intelligence large language models (LLMs), characterized by an extremely high number of parameters. These models employ deep learning to perform language rule modeling, syntactic and semantic parsing, and text generation in natural language processing by analyzing extensive text datasets. Their underlying technology is based on the Transformer architecture, introduced by Vaswani’s team in 2017 (Vaswani et al., 2017), with the self-attention mechanism as a core feature, dynamically assessing text relevance and capturing long-range dependencies, revolutionizing natural language processing and sequence transformation. The integration of large language models into drug discovery represents a paradigm shift in research and development (Zheng et al., 2024). In healthcare, Google’s Med-PaLM model set the bar. Med-PaLM was the first medical LLM to pass the United States Medical Licensing Examination (USMLE), demonstrating its authority in medical question answering tasks. Its iterative version Med-PaLM 2 is based on the more powerful PaLM 2 basic model, and introduces “ensemble optimization” and “retrieval chain” strategies to significantly improve the inference ability. It achieves an accuracy of 86.5% on clinical topic datasets such as MedQA, which is close to or better than the existing optimal level, and performs better in adversarial problem processing and factual accuracy. The multilingual support and generation capabilities of Med-PaLM 2, such as report generation, have been applied in several scenarios such as clinical decision support, health education, and drug discovery. In drug target discovery, these models facilitate literature mining and patent data analysis to explore disease-related biological pathways and core targets. Specialized models trained on biomolecular “language” can analyze and predict multi-omics data, such as genomics, to enhance candidate target identification. Protein language models, including ESMFold (Lin et al., 2023), overcome traditional structural similarity analysis limitations by employing 3D structure prediction technologies. This review systematically examines the innovative applications of large language models in drug target discovery, addressing current technical bottlenecks and development challenges (Figure 1).

This figure summarizes the application of artificial intelligence big language models in drug target discovery and their representative models, including natural language models and language models applied to genomics, transcriptomics, proteomics and multi-omics.

## 2 Analysis of the application of the natural language model in drug target text information mining

Pre-trained language models based on the Transformer architecture, such as GPT and BERT, have gained significant attention in recent years. These models have enabled large-scale advancements in natural language processing due to their strong semantic parsing and text generation capabilities. In biomedical research, they provide innovative solutions for analyzing disease pathogenesis and identifying therapeutic targets through techniques such as literature mining and professional term recognition. The generalization capability of language models pre-trained on extensive text corpora allows for the effective identification of cross-domain linguistic features, while the acquired common language rules significantly improve the efficiency of downstream tasks (Zheng et al., 2024). Biology-specific language models, designed and trained specifically for the biomedical domain, possess an enhanced ability to interpret the semantics of specialized terminology and accurately analyze complex sentence structures and domain-specific concepts within biomedical literature. In drug target design, both general-purpose and domain-specific language models offer unique technical advantages and play an indispensable role in advancing research and development.

### 2.1 General purpose natural language model

General natural language models are trained on extensive text datasets, including scientific papers, textbooks, and general literature. The large-scale training data enable these models to comprehend diverse human languages while also developing a deep understanding of scientific background knowledge. In scientific applications such as drug target discovery, general language models, including GPT-4 (Microsoft Research AI4Science Microsoft Quantum, 2023), DeepSeek, BERT (Devlin et al., 2019), and Claude, can analyze vast amounts of literature, integrate extracted data into knowledge maps, and reveal internal relationships between genes and diseases, enhancing target interpretability. These models contribute to exploring disease mechanisms (Savage, 2023). Their ability to effectively process both complex scientific language and general knowledge provides significant advantages, particularly in terms of broad knowledge coverage and the capability to establish connections across different topics.

### 2.2 Dedicated natural language models

General natural language models have contributed significantly to biomedical text mining. However, these models directly transfer the word distribution of natural language from general corpora to biomedical corpora, limiting their ability to process text requiring specialized biomedical knowledge. With advancements in biomedicine, domain-specific natural language models have been developed. Medical corpora such as PubMed and PubMed Central (PMC) literature are commonly used in the pre-training stage of biomedical-specific language models (Zhou et al., 2023). BERT-derived models, including BioBERT (Lee et al., 2020) and PubMedBERT (Gu et al., 2021), as well as GPT-derived models, such as BioGPT (Luo et al., 2022) and ChatPandaGPT, have enhanced accuracy and efficiency in biomedical natural language processing tasks. BioGPT, developed by Microsoft Research, is based on the GPT architecture and has been optimized through large-scale literature training in the biomedical field. It demonstrates outstanding performance in understanding professional terms and complex conceptual relationships in various multi-task scenarios, such as relation extraction, question answering, and text classification, significantly outperforming its predecessors. Its open-source nature also lowers the barriers for research and application. BioBERT, fine-tuned using data from the Human Protein Atlas, performs multiple functions such as biomedical named entity recognition, relationship extraction, and question answering. Additionally, it extracts information from scientific literature to identify novel drug targets (Lee et al., 2020). ChatPandaGPT, integrated into the PandaOmics platform by Insilico Medicine, facilitates the review of complex data and enables the identification of potential therapeutic targets and biomarkers through natural language interactions. Furthermore, Galactica automatically extracts molecular interactions and pathway information from scientific literature, improving the understanding of complex biological processes and aiding in drug target discovery (Park et al., 2023). DeepSeek also demonstrates potential in biomedical text mining by learning from extensive biomedical literature, enabling precise identification of specialized terminology and complex concepts, thereby providing valuable support for drug target identification. It is worth noting that building high-performance biomedical-specific models typically relies on effective domain adaptation fine-tuning. This is not merely about continuing the pre-training of a general model on biomedical corpora, but rather involves fine-tuning for specific downstream tasks such as relation extraction and target entity recognition. Common strategies include parameter-efficient fine-tuning techniques like LoRA (Low-Rank Adaptation), as well as hierarchical learning rate adjustments, to efficiently inject domain-specific knowledge while retaining general language knowledge and optimizing task performance. This targeted fine-tuning is a key technical approach to overcoming domain shift and improving precision and recall in drug target text mining tasks.

With the capability to parse natural semantics and interpret complex scientific concepts, natural language models serve as a crucial technological tool for enhancing the efficiency of drug target discovery. General-purpose language models offer significant adaptability in multi-task scenarios; however, when applied to specialized domains, optimization through domain-adaptive fine-tuning is often required to improve the accuracy of professional term interpretation and contextual understanding. The primary strength of domain-specific language models lies in their ability to deeply integrate subject-specific knowledge. However, their high specialization limits interdisciplinary applications due to technical constraints. Future advancements are expected to focus on developing hybrid architectures that balance domain-specific accuracy with cross-domain generalization, providing more effective solutions for multidisciplinary research, including biomedicine. It is important to note that current models are predominantly trained on historical literature databases, which may result in the algorithm inheriting biases inherent in human cognition. Moreover, excessive dependence on existing literature data could restrict the model’s capacity for breakthrough innovations in novel drug target discovery. Establishing a multi-dimensional collaborative framework that integrates natural language models, computational biology models, and experimental validation systems may represent a critical technical pathway for identifying innovative and efficient drug targets (Pun et al., 2023; Sarumi and Heider, 2024).

## 3 Analysis of the application of genomics large language model in the discovery of drug target gene code

With the increasing demand for biological data mining in drug target discovery and new drug development, research has expanded the application of natural language processing technology to biological data, which is larger in scale, more complex, and highly specialized. This has led to the emergence of genomics-focused large language models. Genomics primarily investigates an organism’s complete DNA, emphasizing the detailed analysis of genome structure, function, evolution, mapping, and editing. Advancements in next-generation genomic technologies have enabled researchers to generate vast amounts of genomic data (Li R. et al., 2022). The integration of large language models with genomic analysis is now creating new research pathways and application scenarios. Trained on extensive genomic datasets, genome-focused large language models provide deeper insights into gene function, regulation, and interactions while possessing the capability to predict pathogenic variants and gene expression. These capabilities establish a theoretical foundation for drug target discovery and offer strong support for the development of new therapeutic agents.

### 3.1 Gene function prediction

The genomics-focused large language model integrates DNA sequence data to identify functional elements, genetic variations, and structural features, providing a theoretical foundation for drug target discovery. Notable examples include DNABERT (Ji et al., 2021), which transforms DNA sequences into language symbols and utilizes k-mers to capture intricate patterns, enabling high-precision predictions of disease-associated mutations and DNA-protein binding sites. LOGO (Yang M. et al., 2022), a lightweight human genome language model, was developed using pre-trained parameters as initial weights. After task-specific fine-tuning, it has been effectively applied to promoter region identification, enhancer-promoter interaction prediction, chromatin feature inference, and pathogenic variant prioritization. Evo (Nguyen et al., 2024), a multimodal genome infrastructure developed by Arc Institute, facilitates the analysis of natural genomic variations and predicts the effects of small DNA modifications on organism adaptability. This model represents a significant advancement in understanding and designing cross-modal biological systems, establishing a technical foundation for precise target screening.

### 3.2 Regulation of gene expression

Large language models significantly enhance the identification efficiency of core factors involved in gene expression regulation and enable high-precision prediction of gene interaction networks, providing deeper mechanistic insights into gene regulatory network analysis (Joachimiak et al., 2024). The Enformer model, developed by DeepMind, constructs a quantitative prediction framework for enhancer-regulated gene expression by integrating long-range interaction data spanning up to 200,000 base pairs in the genome, exceeding traditional methods by more than fivefold (Avsec et al., 2021). Notably, the progression of various diseases, including tumors, is often associated with epigenetic abnormalities, such as fluctuations in DNA methylation levels and disruptions in histone modifications, which can be targeted through pharmacological interventions. The optimized GeneBERT model, built on the BERT framework, specializes in genome function prediction, effectively inferring the impact of histone modification variations on gene expression and analyzing gene regulatory mechanisms. Additionally, models such as BERT6mA (Tsukiyama et al., 2022), iDNA-ABT (Yu et al., 2021), and MuLan-Methyl (Zeng et al., 2023) facilitate the analysis of DNA sequence methylation characteristics and their potential influence on gene regulatory networks. These advancements have expanded the understanding of how epigenetic modifications regulate gene expression (Sarumi and Heider, 2024), introducing a novel research paradigm for innovative drug target development.

## 4 Analysis of the application of transcriptomics large language model in the construction of drug-target related regulatory network

With advancements in genomic data analysis, research has increasingly shifted toward exploring dynamic gene regulatory systems, emphasizing the systematic study of the spatiotemporal characteristics of gene expression networks and regulatory mechanisms. Transcriptomics, which systematically examines all transcript products within an organism, provides essential data for studying biological processes by analyzing changes in gene expression levels and their functional regulation under various physiological and pathological conditions. This field plays a crucial role in disease mechanism analysis and precision medicine, offering a scientific foundation for optimizing clinical diagnostic and therapeutic strategies and developing personalized medical approaches. Transcriptomics analysis based on large language models facilitates key research tasks, including disease-specific gene expression profiling, gene regulatory network reconstruction, and pathogenic mechanism interpretation. Additionally, it establishes a multi-dimensional data support framework for drug target discovery, enhancing the efficiency and accuracy of identifying potential therapeutic targets.

### 4.1 RNA structure prediction

Structural changes in RNA are often closely associated with its function. Predicting RNA secondary and tertiary structures allows for a deeper understanding of its specific roles in biological processes and facilitates the identification of novel therapeutic targets. RNABERT (Akiyama and Sakakibara, 2022), a pre-trained model based on the BERT architecture, is specifically designed for secondary structure prediction and RNA clustering. This model efficiently aligns unknown sequences with existing RNA families, providing a valuable tool for annotating newly discovered transcripts. RhoFold + integrates the large-scale pre-trained RNA language model RNA-FM to extract sequence features and employs a deep learning module for end-to-end RNA three-dimensional structure prediction, addressing challenges related to data scarcity (Shen et al., 2024). RNA structure prediction not only enhances the understanding of RNA function and binding sites but also serves as a critical structural foundation for drug target discovery and the development of RNA-targeted therapeutics.

### 4.2 Gene expression analysis

In May 2023, the Theodoris research team introduced Geneformer, the first general large language model in the field of transcriptomics (Theodoris et al., 2023). This model was pre-trained using over 30 million single-cell transcriptome datasets and enables three core functions: predicting gene network dynamics, mapping gene networks, and accelerating the identification of therapeutic targets for diseases, even under sparse data conditions (Theodoris et al., 2023). The model was applied to research on hypertrophic cardiomyopathy and dilated cardiomyopathy, successfully screening more than 400 associated genes for each condition. In the case of hypertrophic cardiomyopathy, Geneformer accurately identified specific therapeutic targets and patented drug targets in cardiomyocytes. Additionally, for dilated cardiomyopathy, the inhibition of candidate genes predicted by the model demonstrated an improvement in cardiomyocyte function within disease models. These empirical findings highlight the technical significance of Geneformer in the discovery of therapeutic targets for human diseases.

GeneCompass (Yang et al., 2024), developed by the research team at the Chinese Academy of Sciences, is a cross-species foundational model capable of deciphering gene regulatory codes. It demonstrates significant potential in identifying key factors involved in cell fate regulation and screening candidate drug targets. The Lomics (Wong et al., 2024) model enhances the analysis of complex gene interaction networks by improving the accuracy of pathway analysis and gene set enrichment in transcriptome data, enabling the integrated analysis of multi-omics data. Notably, single-cell large model technologies, such as scBERT (Yang F. et al., 2022) and scFoundation (Hao et al., 2024), also exhibit promising applications in single-cell transcriptome data analysis and related research scenarios. In 2024, it was reported that scFoundation showed “the highest accuracy” in the cell type annotation task, especially in identifying rare cell types such as CD4+ T helper 2 and CD34+ cells.

### 4.3 Post-transcriptional regulatory studies

Post-transcriptional regulation plays a crucial role in controlling gene expression through mechanisms such as RNA splicing, editing, stability regulation, transport, and translation, which are essential for maintaining gene expression homeostasis. The SpliceBERT model (Chen et al., 2024) enhances splice site prediction accuracy, enabling in-depth analysis of splice variant regulatory mechanisms in biological processes and their impact on gene expression. Long non-coding RNA (lncRNA), a key transcription product, significantly influences malignant tumor progression and disease development. Recent studies have identified small open reading frames (sORFs) within lncRNAs capable of encoding functional peptides. The LncCat tool (Feng et al., 2023) is designed to identify lncRNA molecules containing sORFs, providing technical support for discovering novel regulatory elements. RNA modifications are widely involved in life activity regulation and disease evolution. The BERT-m7G system (Zhang et al., 2021) accurately identifies m7G modification sites in RNA sequences, offering a foundation for understanding the regulatory effects of this modification on gene function. By elucidating the dynamic regulatory network of gene expression, post-transcriptional regulation research introduces innovative directions for drug target identification and significantly advances the development of novel therapeutics and personalized medicine.

## 5 Analysis of the application of proteomics big language model in accelerating the prediction of drug target structure and function

While investigating gene regulatory networks, drug target discovery can also be approached by analyzing protein-level characteristics. Proteins play a fundamental role in life processes, serving as key executors of most cellular biological functions. Many diseases are directly associated with the dysfunction of specific proteins. Through an in-depth examination of protein structure, function, and interactions, disease-related targets can be precisely identified, facilitating the development of highly specific and effective drugs. By learning and analyzing protein sequence, structure, and omics data, large language models have demonstrated significant potential in accelerating data analysis, enhancing drug target screening and design, and improving structure prediction. These advancements not only increase research efficiency but also contribute to reducing overall research costs.

### 5.1 Protein structure prediction

The three-dimensional conformation of proteins plays a crucial role in drug target identification, as the specific binding of drug molecules to target proteins typically relies on precise structural compatibility. As of 2025, the UniProt database contains over 250 million protein sequences, while the PDB database holds only about 240,000 3D structures covering about 70,000 proteins, representing less than 0.1% of known proteins. Traditional experimental techniques for protein structure analysis are time-intensive and costly, creating a significant gap between output efficiency and the demands of drug research and development (Dana et al., 2019). In recent years, deep learning and artificial intelligence technologies have transformed protein structure prediction. A major breakthrough was achieved in 2020 with the development of AlphaFold2 by DeepMind. Utilizing a homologous sequence alignment strategy, this model reached near-experimental accuracy. Following the prediction of over 200 million protein structures, about 35% met high-confidence criteria, and 80% of the structural data exhibited multi-dimensional characteristics. By integrating an attention mechanism with a self-distillation training strategy, the model significantly enhances predictive performance, advancing the field of protein structure analysis and its applications in drug discovery. RoseTTAFold, introduced alongside AlphaFold2, employs a three-track attention architecture that enables neural networks to process three-dimensional information simultaneously, establishing a technical benchmark comparable to AlphaFold2. In 2022, Andrew G. Jamieson’s research group utilized the AlphaFold-predicted structure of the GPR84 protein to conduct a structure-activity relationship study on GPR84 antagonists. This study identified compounds 7 and 8 as exhibiting favorable activity and selectivity, validating the utility of AI-predicted structures in target optimization (Marsango et al., 2022; Mahin et al., 2022). On 31 October 2023, AlphaFold3, jointly developed by DeepMind and Isomorphic Labs, introduced a diffusion module to replace the original structure module, reducing reliance on homologous sequences. This model achieves high-accuracy predictions of protein interactions with various biomolecules, improving overall accuracy by more than 50% compared to its predecessor and achieving a twofold breakthrough in key performance metrics. Recent studies integrating AlphaFold3 with Mendelian randomization methods successfully identified seven proteins with structural abnormalities resulting from missense mutations, providing new insights into the mechanisms of Alzheimer’s disease and facilitating the screening of potential therapeutic targets.

The field of protein structure prediction is undergoing a paradigm shift, with protein language models based on direct sequence prediction offering advantages in both computational efficiency and accuracy. RGN2, introduced in 2022, employs an end-to-end microcyclic geometric network architecture, where the AminoBERT protein language model extracts potential structural features from unaligned sequences. This approach has demonstrated superior performance over traditional homology alignment methods from both practical and theoretical perspectives. Simultaneously, Meta released the ESMFold model, built on the Transformer framework with 15 billion parameters, capable of predicting amino acid sequence structures with atomic-level accuracy. This model achieves a computational speed ten times faster than AlphaFold2 while maintaining comparable prediction accuracy, enabling large-scale real-time resolution of metagenomic protein structures. The average GDT_TS score of ESMFold on CASP15 was 61.62, close to the 73.06 of AlphaFold2, indicating good performance but still a gap. ESMFold, on the other hand, predicted shorter amino acid sequences an order of magnitude faster than AlphaFold2, even 60 times faster in some cases. Its subsequent version, ESM2, further set the bar for protein language models. ESM2 is also based on the Transformer architecture and focuses on efficient generation of semantic representations of protein sequences. It performs well in protein structure prediction, function annotation (e.g., GO classification, BP function prediction), and drug target identification (e.g., TCR-pMHC interaction prediction). Compared with models such as ProtT5, ESM2 is particularly outstanding in terms of speed and performance balance. Its simple “no external encoder” design simplifies the protein processing process and provides a powerful new tool for protein engineering and target discovery. These advancements highlight the capability of language models to identify evolutionary patterns and structural features from extensive sequence data, establishing a robust foundation for reverse molecular docking and binding site similarity analysis in structural biology.

### 5.2 Protein sequence generation

The advancement of big data and artificial intelligence technologies has introduced an innovative approach to target discovery—a new paradigm centered on protein sequence generation. The ProGen2 model (Nijkamp et al., 2023) generates novel protein sequences with predefined structural and functional properties by analyzing complex sequence patterns and their interrelations. ProtGPT2 (Ferruz et al., 2022), developed using the GPT-2 autoregressive architecture, enhances protein engineering design and function prediction capabilities. The generated sequences exhibit compositional tendencies that align with the distribution characteristics of natural amino acids. These AI-generated proteins not only adhere to the principles of biological evolution but can also be tailored to exhibit specific functional properties, providing a valuable tool for identifying potential targets in previously unexplored biological domains. Molecular docking simulation technology allows researchers to virtually screen these AI-generated sequences against existing drug libraries, enabling the selection of candidate proteins with high binding affinity, followed by targeted experimental validation studies.

### 5.3 Protein function prediction

Proteins serve as core functional modules in regulating cellular metabolism, signal transduction networks, and structural support systems. Therefore, systematic analysis of protein biological functions holds significant scientific value for drug target discovery and disease mechanism research (Liu et al., 2024). The ProteinBERT model (Brandes et al., 2022) efficiently captures complex sequence features and biological characteristics by processing large-scale sequence data, demonstrating strong versatility across various protein research tasks. The ProtST framework (Xu et al., 2023), a multimodal learning system for protein sequences and biomedical texts, integrates sequence data with textual information to enhance feature representation and enable in-depth protein function analysis. This model facilitates the identification of functional proteins from extensive databases, even in cases lacking functional annotation. ESM-1b applies a self-supervised learning strategy to process vast numbers of unlabeled sequences, effectively extracting evolutionarily conserved features and residue interactions. The QuoteTarget method (Chen et al., 2023) innovatively combines ESM-1b with a graph convolutional neural network to achieve efficient protein coding using only sequence information. This approach achieved 95% classification accuracy on a non-redundant drug target validation dataset. When applied to the entire human proteome, it successfully identified 1,213 previously unexplored potential therapeutic targets.

Intrinsic disordered regions (IDRs) represent a distinct class of domains within protein sequences, characterized by conformational dynamics and the absence of a stable three-dimensional structure under physiological conditions. The structural flexibility of IDRs allows them to interact with various ligand molecules, making them highly valuable sites for drug action. Accurate identification of IDRs and elucidation of their functional mechanisms are crucial for enhancing drug design efficiency (Pang and Liu, 2024). The DisoFLAG model (Pang and Liu, 2024), a specialized prediction tool for disordered regions, employs a sequence-driven strategy to precisely locate IDRs and analyze their functional properties. As an emerging target in drug development, IDRs may provide a novel pathway for therapeutic discovery. In drug-target interaction studies, accurate assessment of binding affinity is a critical step in the research and development process. Traditional experimental methods face limitations in scalability, leading to the development of various computational prediction models, including sequence-driven approaches, graph neural networks, and multimodal fusion techniques. While these methods have shown progress, further improvements in prediction accuracy and mechanistic interpretability remain necessary.

### 5.4 Antigen-receptor interaction and recognition of neoantigens

In the study of cancer, immune system diseases, and infectious diseases, a comprehensive understanding of the interaction mechanisms between antigens and receptors may contribute to the advancement of drug target development. These fundamental research breakthroughs also provide new opportunities for the implementation of personalized medical strategies. In China’s current medical innovation framework, malignant tumor research holds a dominant position in clinical trials and drug development (Li et al., 2022b). Neoantigens, which serve as specific tumor markers, arise from gene mutations or abnormal alterations in genetic material within cancer cells. These antigens are entirely absent in healthy tissues. Due to their unique tumor-specific properties, neoantigens can effectively activate the immune response, making them a focal point in immunotherapy research (Li et al., 2023). For example, a team from Peking University Cancer Institute successfully used a neoantigen prediction model combined with TCR sequencing to screen highly immunogenic neoantigens for specific solid tumor patients and used them for individualized TCR-T cell therapy. The initiation of an immune response relies on the binding of antigen peptides to T cell receptors (TCR), a process mediated by major histocompatibility complex (MHC) molecules. As the central recognition element of T lymphocytes in identifying pathogens and malignant cells, TCR plays a crucial role in the immune defense mechanism. For the quantitative assessment of the binding affinity between MHC-I and MHC-II molecules and peptides, prediction models such as MHCRoBERTa (Wang F. et al., 2022) and BERTMHC (Cheng et al., 2021) have been developed, significantly enhancing the efficiency of predicting interactions between key immune molecules. Based on the BERT framework, the TCR-BERT model (Wu et al., 2024) enables the analysis and prediction of TCR-antigen interactions using deep learning technology. This approach not only expands the analytical scope of antigen recognition but also allows for more precise and adaptable characterization of binding properties. Notably, the complementarity-determining region 3 (CDR3) of TCR molecules serves as a functional domain directly involved in antigen contact, with its sequence variation being a critical determinant of TCR receptor diversity. The TCR-BERT model effectively extracted generalized feature representations of TCR sequences by training on extensive unlabeled CDR3 sequence data, and this pre-training strategy significantly improved the performance of subsequent antigen-specific recognition prediction models (Liu et al., 2024).

### 5.5 Large language models of antibodies

In the advancement of immunology and biomedicine, large language model-based technologies are increasingly demonstrating their significance. These models, inspired by protein language models, enable the prediction of key parameters such as antibody structural features, functional activity, interaction dynamics, and binding affinity. By deciphering the “language rules” of antibodies, AntiBERTa can trace the developmental origins of B-cell-derived antibodies, evaluate immunogenicity strength, and predict potential binding sites and other complex tasks (Leem et al., 2022). As a deep learning-based epitope prediction tool, ParaAntiProt efficiently extracts predictive feature embeddings by integrating pre-trained protein and antibody language models. This approach requires only amino acid sequence data, eliminating dependence on antigen-related information (Kalemati et al., 2024). Due to the unique gene rearrangement mechanism and the diversity of complementarity-determining regions, antibody three-dimensional structure prediction presents distinct challenges in protein structure research. The pre-trained IgFold model (Ruffolo et al., 2023), trained on a dataset of 558 million natural antibody sequences, achieves atomic coordinate prediction with accuracy comparable to AlphaFold while offering significant advantages in computational efficiency. As an advanced iteration, AlphaFold3 has introduced breakthroughs in antibody structure prediction, particularly in the structural modeling of the heavy chain complementarity-determining region 3 (CDR H3), a critical domain that defines antigen binding specificity and affinity. This model has markedly reduced the root mean square deviation of predictions from 2.74 Å to 1.34 Å (Abramson et al., 2024). The application of large language models has greatly enhanced the efficiency and optimization of antibody design, providing essential technical support for novel drug target development. Additionally, these advancements establish a crucial foundation for precision medicine, vaccine development, and improvements in antibody-based therapeutics.

### 5.6 LLM-driven automated drug molecule design

After successfully identifying and validating protein targets and their key features (such as binding sites and functional domains), one of the core challenges in drug development lies in efficiently designing candidate drug molecules that can effectively act on these targets. Large language models (LLMs) are being utilized to build end-to-end automated molecular design systems, significantly accelerating this process. Take the DrugAgent system as an example. As a multi-agent framework based on LLMs, it aims to automate key steps in the drug discovery process, including data acquisition, model training, result evaluation, and final molecular design optimization, achieving an end-to-end design from target information to candidate molecules. The core of this system is driven by two agent components: the LLM Director is responsible for integrating professional knowledge from fields such as drug chemistry, biology, and pharmacology to guide the direction of the entire molecule generation and optimization process; the LLM Planner is responsible for optimizing the search strategy in the molecular space and dynamically adjusting the generation direction based on experimental feedback or prediction results. In empirical studies, the model guided by the DrugAgent system achieved significant performance improvements in predicting the intestinal absorption characteristics of drug molecules (based on the PAMPA dataset), with an F1 score reaching 0.92, significantly outperforming traditional methods, effectively demonstrating the effectiveness of LLMs in integrating knowledge, guiding model selection, and optimizing the prediction process. The technical highlight of this system lies in its dynamic idea space management capability, which can generate structurally diverse molecules in the early exploration stage to expand the coverage of the chemical space; and it builds an iterative optimization closed loop, continuously improving the quality of generated molecules based on experimental data or computational prediction feedback (such as absorption, activity, toxicity, etc.). Ultimately, through the automatic coordination of data, models, and decisions by the agents, it significantly reduces manual intervention and lowers the reliance on the highly expert-experience-dependent and manual operation-intensive steps in traditional drug design, significantly enhancing overall efficiency. The DrugAgent system is a typical representative of the LLM-enabled “rapid design” paradigm for drug molecules. Its core lies in constructing a highly automated “design-predict-verify (or simulate)-redesign” closed loop. The LLM Planner can adjust the search strategy and generation direction in real time based on feedback (whether from experiments or high-precision computational simulations, such as molecular docking and ADMET prediction models), for example, focusing on specific chemical spaces, avoiding known toxic groups, and optimizing specific properties. This dynamic, data-driven iterative process greatly accelerates the cycle of lead compound discovery and optimization, significantly differentiating from the time-consuming and labor-intensive trial-and-error exploration in traditional methods. By combining expert knowledge (encoded in the LLM Director) with automated iterative optimization, such systems can explore the vast chemical space at a speed far exceeding traditional methods, achieving rapid discovery and evolution of candidate molecules.

## 6 Analysis of the application of single-cell multi-omics large language model in the integration of multi-dimensional data for drug target discovery

Across multiple research domains, including genomics, transcriptomics, and proteomics, large-scale language models facilitate targeted analysis and provide critical insights for drug target screening. Notably, single-cell multi-omics language models significantly broaden the scope of target identification by integrating multi-dimensional data, enabling the discovery of potential therapeutic targets that may not be captured using traditional methods. As systems medicine continues to drive improvements in drug research and development efficiency, multi-omics technology has emerged as a key enabler (Wang M. et al., 2022). By collaboratively analyzing biological data across genomics, transcriptomics, proteomics, and metabolomics, researchers can perform cross-comparisons and in-depth assessments of multi-source omics data. This approach allows for precise identification of disease-associated signaling pathways and core regulatory elements, ultimately leading to the selection of candidate targets with therapeutic potential. This systematic research framework not only enhances the understanding of disease mechanisms but also provides a scientific foundation for drug molecular design and optimization of therapeutic efficacy, achieving the dual objectives of improving treatment outcomes while minimizing adverse effects.

**FIGURE 2:**
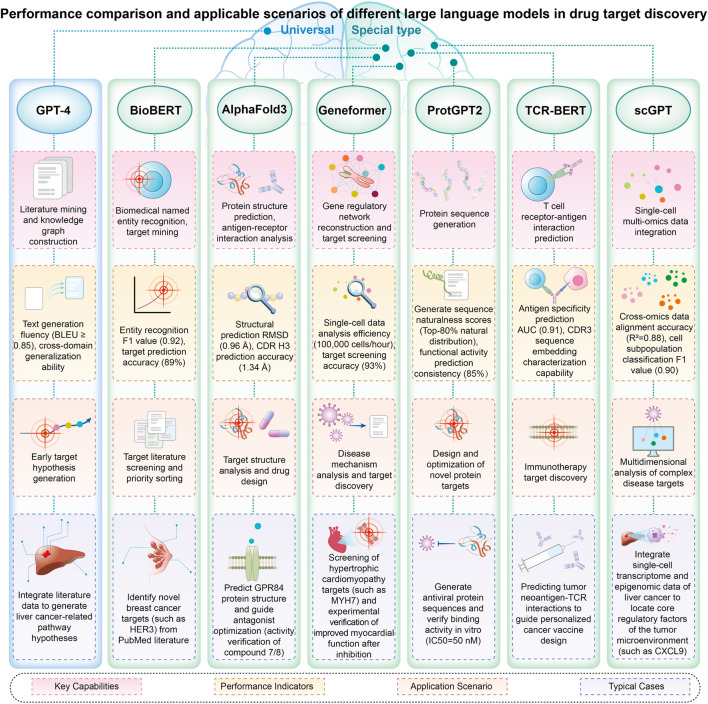
Performance comparison and applicable scenarios of different large language models in drug target discovery.

The application of large language models in multi-omics data integration is demonstrating significant breakthroughs. The scGPT model (Cui et al., 2024), leveraging single-cell multi-omics data, enables in-depth analysis of gene interactions at single-cell resolution through the collaborative examination of genetic regulatory networks, enhancing the biological interpretability of the model. The scMVP system (Li et al., 2022c) is an innovative framework specifically designed for the integrated analysis of single-cell transcriptome sequencing (RNA-seq) and epigenome sequencing (ATAC-seq) data, facilitating simultaneous examination of gene expression patterns and chromatin accessibility within individual cells. For single-cell multimodal data, including RNA sequencing, ATAC sequencing, and antibody marker sequencing, the DeepMAPS model (Ma et al., 2023) successfully establishes the mapping relationship between cell subtypes and gene functional modules by constructing a gene-cell two-dimensional network, enabling the collaborative learning of both local and global features. By incorporating high-precision multi-dimensional data, single-cell multi-omics research offers innovative solutions to address challenges such as data variability, sample dispersion, and cell subset diversity (Liu et al., 2024) (Figure 2).

## 7 Summary and outlook

Drug development, a time-intensive and costly process, prioritizes drug target discovery, which involves identifying biomolecules or regulatory pathways that play a critical role in disease mechanisms. However, due to the high complexity and technical challenges in this field, the number of validated effective drug targets remains limited. In recent years, advancements in experimental technology, multi-omics analysis platforms, and computational methods have significantly contributed to the refinement of target identification strategies. Despite these advancements, traditional experimental approaches and multi-omics studies often require substantial resources, and the reliability of results is highly dependent on the standardization of biological samples. Artificial intelligence technology, particularly models based on the Transformer architecture, is reshaping the drug research and development landscape. These models achieve human language parsing and logical text generation through deep learning from large-scale text datasets. In drug target design, natural language processing enables systematic literature mining and the construction of patent maps, enhancing the efficiency and accuracy of target discovery. Specialized models such as BioBERT significantly enhance the accuracy and efficiency of biomedical text processing by precisely analyzing scientific concepts. Notably, large language models have demonstrated breakthrough potential in genome analysis, transcriptional regulation research, protein system analysis, and single-cell multi-omics integration. In genomics, dedicated models have deepened the understanding of gene function, regulatory mechanisms, and interactions while significantly improving the prediction of pathogenic mutations and inference of expression patterns. In transcriptomics, tools such as Geneformer simulate dynamic changes in gene networks, providing a multi-dimensional biological foundation for drug target discovery. Protein language models play a crucial role in structure-activity relationship prediction, target screening, and optimization, continuously improving research efficiency and reducing development costs. Single-cell multi-omics integration models effectively analyze disease-related signaling pathways and core regulatory elements by integrating multi-dimensional data. These technological advancements not only enhance the understanding of fundamental biological processes but also accelerate target identification and drug development, paving the way for personalized medicine.

Although artificial intelligence has achieved significant advancements in target discovery, several challenges remain in its practical application. A major limitation is the need to improve the interpretability of algorithmic predictions, which is essential for gaining acceptance in scientific research and medical fields. Current models primarily focus on sequence features and functional analysis of gene expression regulatory networks. However, limited consideration of critical parameters such as target specificity, tissue distribution characteristics, and toxicological properties may reduce the translational value of prediction results. Strengthening these aspects is crucial for enhancing the reliability and applicability of AI-driven target discovery in drug development. Data bias presents a significant challenge in model training, as models developed using biased datasets may reinforce inherent cognitive biases (Sarumi and Heider, 2024). Addressing this issue requires the construction of diverse and representative training datasets to enhance model generalization. Additionally, in the extensive data collection and model development processes, ethical review, privacy protection, and compliance framework establishment remain critical areas for improvement (Pun et al., 2023). Going forward, establishing a unified, comprehensive and challenging Benchmarking system is essential to promote the development of LLM in the field of drug target discovery. Currently, the data sets, evaluation metrics, and comparison baselines used by different research teams vary greatly, making it difficult to objectively compare model performance. There is an urgent need to build standardized benchmark datasets and evaluation protocols covering the whole process of target discovery, from literature knowledge mining, omics data analysis, target property prediction to molecule generation and optimization. These benchmarks should incorporate publicly available gold standard datasets (e.g., known validated targets, protein-ligand complex structures, activity/toxicity data), negative samples (nontarget/inactive molecules), and tasks that mimic real-world complexity (e.g., predicting novel targets, processing noisy data, generalizing across tissues/diseases). At the same time, the evaluation index should go beyond the simple accuracy or AUC, and should include the comprehensive consideration of the biological rationality, interpretability, computational efficiency and finally the impact on the success rate of experimental verification. Strong benchmarking will facilitate iterative model optimization, fair comparison of different technology routes, and ultimately drive the establishment of best practices in the field. With continuous technological advancements, large language models are expected to further expand their applications in drug research and development, accelerating progress through innovative analytical paradigms. These advancements will enhance the efficiency, innovation, and cost-effectiveness of drug target discovery and new drug development, ultimately driving a transformative shift in the pharmaceutical industry.

## Abbreviations

DOAJ, Directory of open access journals; LD, Linear dichroism; MDPI, Multidisciplinary Digital Publishing Institute; TLA, Three letter acronym.
